# Overview of Veno-Arterial Extracorporeal Membrane Oxygenation (VA-ECMO) Support for the Management of Cardiogenic Shock

**DOI:** 10.3389/fcvm.2021.686558

**Published:** 2021-07-07

**Authors:** Adamantios Tsangaris, Tamas Alexy, Rajat Kalra, Marinos Kosmopoulos, Andrea Elliott, Jason A. Bartos, Demetris Yannopoulos

**Affiliations:** ^1^Division of Cardiology, Department of Medicine, University of Minnesota, Minneapolis, MN, United States; ^2^Center for Resuscitation Medicine, University of Minnesota School of Medicine, Minneapolis, MN, United States

**Keywords:** extracorporeal membrane oxygenation, cardiogenic shock, mechanical circulatory support, VA-ECMO indications, VA-ECMO complications

## Abstract

Cardiogenic shock accounts for ~100,000 annual hospital admissions in the United States. Despite improvements in medical management strategies, in-hospital mortality remains unacceptably high. Multiple mechanical circulatory support devices have been developed with the aim to provide hemodynamic support and to improve outcomes in this population. Veno-arterial extracorporeal membrane oxygenation (VA-ECMO) is the most advanced temporary life support system that is unique in that it provides immediate and complete hemodynamic support as well as concomitant gas exchange. In this review, we discuss the fundamental concepts and hemodynamic aspects of VA-ECMO support in patients with cardiogenic shock of various etiologies. In addition, we review the common indications, contraindications and complications associated with VA-ECMO use.

## Introduction

The primary objective of this paper is to provide a comprehensive review of veno-arterial extracorporeal membrane oxygenation (VA-ECMO) use in the management of adult patients with refractory cardiogenic shock (CS).

## The Evolving Definition of Cardiogenic Shock

Cardiogenic shock is commonly defined as a state of low cardiac output that is inadequate to support the systemic perfusion requirements in the context of normal cardiac filling pressures. Organ hypoperfusion is a central feature of CS. The resultant tissue ischemia and reduced nutrient delivery, if persistent, may lead to multi-organ failure including altered mental status, oliguria with <30 cc/h urine output, narrow pulse pressure, and arterial lactic acid level exceeding 2 mmol/L (1, 2).

Historically, clinicians and investigators established the presence of CS by using a combination of select abnormal hemodynamic parameters and evidence of end-organ dysfunction. Consequently, various landmark clinical trials employed different definitions to diagnose CS

(Table 1). Most commonly using some combination of the following criteria: (I) profound hypotension with a systolic blood pressure (BP) <80–90 mmHg for at least 30 min, a drop in mean BP of 30 mmHg or more from baseline, the need for vasoactive medications to maintain a systolic BP above 90 mmHg despite adequate fluid resuscitation; (II) elevated biventricular filling pressures with central venous pressure (CVP) above 10 mmHg and pulmonary capillary wedge pressure (PCWP) exceeding 15 mmHg; (III) significantly reduced cardiac index (<1.8 L/min/m^2^ or <2.2 L/min/m^2^ with hemodynamic support); and (IV) low mixed venous blood oxygen saturation signaling increased peripheral oxygen extraction due to hypoperfusion (16). Systemic vascular resistance (SVR) is markedly elevated in most cases of CS. While calculating SVR is critical to establish the type of shock in routine clinical practice, it has not been included in the definition of CS used by landmark clinical trials as patients may initially present with normal or even low SVR. The presence of low SVR may signify end-stage CS as a result of inappropriate vasodilation despite hypotension, low cardiac output, and tissue hypoperfusion. Accordingly, it is associated with microvascular dysfunction, more severe systemic inflammatory response (cytokine storm) and, ultimately, worse clinical outcomes (22). Coronary perfusion pressure and, therefore, coronary blood flow may decrease significantly in CS owing to the severely elevated ventricular filling pressures and systemic hypotension. This will further worsen myocardial ischemia and contractility contributing to the vicious cycle of CS (8, 18).

**Table 1:** The broad range of criteria utilized to define cardiogenic shock.

**Study**	**Definition**
Aissaoui et al. USIK/UCIC/FAST-MI registries (3)	•SBP < 90 mmHg •Oliguria or signs of peripheral hypoperfusion
Basir et al. The Detroit cardiogenic shock initiative (4)	•SBP < 90 mmHg or need for supportive measures to maintain SBP > 90 mmHg •Signs of peripheral hypoperfusion or oliguria or elevated lactate •Cardiac index < 2.2 LPM/m^2^ or PCWP ≥ 15 mmHg
Bisdas et al. (5)	•SBP < 90 mmHg •Lactate ≥ 4 mmol/L •Cardiac index < 2.2 LPM/m^2^
Brechot et al. (6)	•LVEF < 25% or increased inotrope score or SBP < 90 mmHg despite inotrope use •Cardiac index < 2.2 LPM/m^2^
Brechot et al. (7)	•LVEF < 35% •Lactate ≥ 4 mmol/L •Cardiac index < 3 LPM/m^2^
Califf et al. (8)	•SBP < 90 mmHg for more than 30 min or SBP drop >30 mmHg from baseline for 30 min •Cardiac index < 2.2 LPM/m^2^ or PCWP ≥ 15 mmHg •Oliguria, signs of peripheral hypoperfusion or avO_2_ > 5.5 ml/dL
Chioncel et al. ESC heart failure long-term registry (9)	•SBP < 90 mmHg or drop > 30 mmHg from baseline for 30 min •Oliguria or signs of peripheral hypoperfusion
Chung et al. (10)	•SBP < 90 mmHg and pulmonary edema or need for supportive measures to maintain SBP > 90 mmHg
De Roo et al. (11)	•MAP ≤ 60 mmHg •Cardiac index < 2.2 LPM/m^2^ with hemodynamic support
Goldberg et al. (12)	•SBP < 80 mmHg •Signs of peripheral hypoperfusion or oliguria
Goldberg et al. (13)	•SBP < 80 mmHg •Signs of peripheral hypoperfusion or oliguria
Harjola et al. CardShock study (14)	•SBP < 90 mmHg for more than 30 min or need for supportive measures to maintain SBP > 90 mmHg •Signs of peripheral hypoperfusion or lactate ≥ 2 mmol/L
Helgestad et al. (15)	•SBP < 90 mmHg for more than 30 min or need for supportive measures to maintain SBP >90 mmHg •Signs of peripheral hypoperfusion, oliguria or lactate ≥ 2.5 mmol/L
Hochman et al. SHOCK study (16)	•SBP < 90 mmHg for more than 30 min or need for supportive measures to maintain SBP > 90 mmHg •Cardiac index < 2.2 LPM/m^2^ or PCWP ≥ 15 mmHg •Oliguria or signs of peripheral hypoperfusion
Hochman et al. SHOCK study (17)	•SBP < 90 mmHg for more than 30 min or need for supportive measures to maintain SBP > 90 mmHg •Signs of peripheral hypoperfusion or oliguria •Cardiac index < 2.2 LPM/m^2^ or PCWP ≥ 15 mmHg
Hollenberg et al. (18)	•SBP < 90 mmHg for more than 30 min •Cardiac index < 2.2 LPM/m^2^ or PCWP ≥ 15 mmHg
Holmes et al. GUSTO-I (19)	•SBP < 90 mmHg for more than 60 min or need for supportive measures to maintain SBP > 90 mmHg •PCWP ≥ 15 mmHg
Hulman et al. (20)	•Cardiac index < 2 LPM/m^2^ with support
Killip et al. (21)	•SBP < 90 mmHg •Oliguria or signs of peripheral hypoperfusion
Kohsaka et al. SHOCK study (22)	•SBP < 90 mmHg for more than 30 min or need for supportive measures to maintain SBP > 90 mmHg •Cardiac index < 2.2 LPM/m^2^ or PCWP ≥ 15 mmHg •Oliguria or signs of peripheral hypoperfusion
Lee et al. (23)	•SBP < 90 mmHg for more than 30 min or need for supportive measures to maintain SBP > 90 mmHg
Muller et al. ENCOURAGE derivation cohort (24)	•LVEF < 25% or SBP < 90 mmHg despite inotrope use •Cardiac index < 2.2 LPM/m^2^
Ostadal et al. ECMO-CS (25)	•LVEF < 35% or LVEF 35–55% in combination with valvular disease or need for supportive measures to maintain MAP > 50 mmHg •Cardiac index < 1.8 LPM/m^2^ without support or central venous pressure >7 mmHg or PCWP ≥ 12 mmHg •SvO_2_ < 50% in two consecutive measurements
Ouweneel et al. (26)	•SBP < 90 mmHg for more than 30 min or need for supportive measures to maintain SBP > 90 mmHg
Pozzi et al. (27)	•SBP < 90 mmHg •Signs of peripheral hypoperfusion or oliguria
Rihal et al. SCAI/ACC/HFSA/STS guidelines on MCS use for cardiogenic shock (28)	•SBP < 90 mmHg for more than 30 min or drop >30 mmHg from baseline for 30 min •Cardiac index < 2.2 LPM/m^2^ with support or cardiac index < 1.8 LPM/m^2^ without support or PCWP ≥ 15 mmHg
Seyfarth et al. ISAR-SHOCK (29)	•SBP < 90 mmHg for more than 30 min or need for supportive measures to maintain SBP > 90 mmHg •Signs of peripheral hypoperfusion or oliguria •Cardiac index < 2.2 LPM/m^2^ or PCWP ≥ 15 mmHg
Sheu et al. (30)	•SBP < 90 mmHg and pulmonary edema or need for supportive measures to maintain SBP > 90 mmHg
Thayer et al. Cardiogenic shock working group registry (31)	•SBP < 90 mmHg for more than 30 min •Cardiac index < 2.2 LPM/m^2^
Thiele et al. (32)	•SBP < 90 mmHg for more than 30 min or need for supportive measures to maintain SBP > 90 mmHg •Oliguria •Cardiac index < 2.2 LPM/m^2^ with support or cardiac index < 1.8 LPM/m^2^ without support or PCWP ≥ 18 mmHg
Tsao et al. (33)	•SBP < 90 mmHg and pulmonary edema or intervention required to maintain SBP > 75 mmHg
Wu et al. (34)	•Refractory ventricular tachycardia or need for supportive measures to maintain SBP > 90 mmHg

*SBP, systolic blood pressure; MAP, mean arterial pressure; PCWP, pulmonary capillary wedge pressure; LVEF, left ventricular ejection fraction; SvO2, mixed venous blood oxygen saturation; avO2, arteriovenous oxygen difference; SCAI, Society for Cardiovascular Angiography and Interventions; ACC, American College of Cardiology; HFSA, Heart Failure Society of America; STS, Society of Thoracic Surgeons; MCS, mechanical circulatory support; CS, cardiogenic shock*.

Up until recently, the diagnosis of CS was binary (present or absent) and was established based on a combination of distinct hemodynamic parameters detailed above. However, it became increasingly clear that the clinical condition of patients meeting the minimum criteria of CS are extremely heterogenous. It may include outpatients with low cardiac output, those requiring a single inotrope infusion as well as end stage patients needing biventricular mechanical circulatory support (MCS). Recognizing the continuum of hemodynamic instability in this population, the Society for Cardiac Angiography and Intervention (SCAI) recently published an expert consensus statement defining five stages of CS ranging from at risk to extremis (35, 36). A combination of easily identifiable hemodynamic parameters, biochemical markers and physical examination findings define each stage. This simple and validated framework aims to facilitate targeted patient management by matching the intensity of medical therapy and the level of mechanical support to each individual's CS stage. In addition, physicians can quickly and frequently re-assess their patient's CS stage and adjust the management accordingly. Utilizing this strategy is expected to reduce complications, improve clinical outcomes, and survival.

## Epidemiology of Cardiogenic Shock

Accurately pinpointing the prevalence of CS is challenging and varies based on the era the data was collected and the definition used (21, 35, 37, 38). CS is estimated to account for ~100,000 annual hospitalizations in the United States alone (12, 13, 19, 32). Various studies and randomized clinical trials focusing on patients with myocardial infarction (MI) have reported a prevalence of 6–10%, with a slight increase over time (19, 39–42).

Acute coronary syndrome is the most frequent cause of CS, representing 80% of all cases (14). While more common in patients with ST-elevation myocardial infarction (STEMI), it may also complicate non-STEMI (43, 44). Prior to the advances in medical therapy and interventional strategies, in-hospital mortality of post-MI CS reached 80% with nearly half of these occurring within the first 24-h of presentation (9, 21). Although the emphasis on optimal medical therapy, MCS use and the widespread adoption of early revascularization strategies led to a significant decline in mortality rates, the mortality of CS associated with ACS remains high at 30–50% (3, 13, 45–47). Elderly patients (age >75 years), females, and those with underlying diabetes mellitus or prior myocardial injury are particularly at risk.

While the incidence of post-MI CS has declined over the past decades, there has been a concomitant increase in the incidence of CS caused by other etiologies (48). The most common causes include acute on chronic heart failure (HF), fulminant myocarditis, high-risk pulmonary embolism, stress-induced cardiomyopathy, severe valvular disease, sepsis, and hemodynamically unstable arrhythmias (2, 14). Among ~8 million HF hospitalizations between 2005 and 2014 recorded in the National Inpatient Sample, the incidence rose from 4.1 to 15.6 per one thousand HF hospitalizations (48). For the same time period, a large registry analysis found that the proportion of patients admitted with post-MI CS has dropped significantly from 65.3 to 45.6% (49). The overall in-hospital mortality rate for this population initially was 42.4% but has decreased substantially to 27.1% (49).

## Mechanical Circulatory Support Strategies for the Management of Cardiogenic Shock

Multiple MCS devices have been developed over the past decades with the aim to provide various levels of hemodynamic support to improve the devastating morbidity and mortality associated with CS. The fundamental assumption is that ventricular support and decompression leads to a reduction in myocardial wall stress and oxygen consumption, while concurrently augmenting end organ perfusion.

Several types of MCS devices are used in routine clinical practice. The intra-aortic balloon pump (IABP) was first developed in the 1960s and remains the most frequently utilized percutaneous temporary MCS device (50). While it only provides a modest increase in cardiac output, it augments diastolic coronary flow and reduces myocardial oxygen consumption (28). Newer percutaneous ventricular assist devices (pVAD) can provide significantly higher level of hemodynamic support and include the Tandemheart (LivaNova, London, UK) and the Impella family (Abiomed Inc., Danvers, MA, US). The increasingly utilized VA-ECMO systems (Centrimag, Abbott, Chicago, IL, US and Cardiohelp, Maquet, Rastatt, Germany) provide complete hemodynamic support and concomitant gas exchange. Randomized clinical trials directly comparing the efficacy and outcomes achieved with these devices are scarce and are limited by low enrollment, the predominance of post-MI patients and the highly variable definition of CS (26, 29, 51, 52) (Table 1).

## Introduction to VA-ECMO

VA-ECMO is a temporary mechanical circulatory support system that enables complete and immediate cardiopulmonary support in the setting of cardiogenic shock and cardiac arrest (53). It consists of a centrifugal pump capable of propelling up to 8 L/min of blood and venous drainage and arterial return cannulas. A hollow fiber membrane oxygenator is spliced into the circuit that not only provides blood oxygenation but also carbon dioxide (CO_2_) clearance via sweep gas flow. This latter function is a critical distinguishing feature from other MCS strategies, such as IABP and pVADs. VA-ECMO may also be placed surgically, especially in the post-cardiotomy setting, when oxygenated blood is returned directly into the ascending aorta (central cannulation technique). However, this review focuses primarily on the use of peripherally placed VA-ECMO as this is the most common type of support instituted by cardiologists in the setting of cardiac arrest or refractory CS.

The preferred approach for percutaneous VA-ECMO is femoral artery and vein cannulation. In an adult, the tip of an 18–28 Fr cannula draining deoxygenated venous blood is positioned in the mid right atrium (RA) or the superior vena cava-RA junction. After passing through the “membrane lung,” oxygenated blood is returned to the systemic circulation via a 15–19 Fr arterial cannula with its tip typically positioned in the iliac artery. Selecting cannulas with appropriate diameters is critical not only to reduce the risk of vascular injury but also to avoid significant negative inflow (preferably <50 mmHg) and high outflow pressure (<300 mmHg). To mitigate the risk of distal limb ischemia, an 8 Fr distal reperfusion cannula is routinely inserted into the superficial femoral artery in our center and is spliced into the arterial limb of the circuit (2, 54).

Peripheral VA-ECMO is increasingly utilized as a short-term support strategy to manage patients presenting with cardiac arrest, severe biventricular HF and CS stages C-E, independent of etiology (48). It can be initiated safely in the cardiac catheterization laboratory by experienced interventional cardiologists with very short door to support time, even during ongoing cardiopulmonary resuscitation (CPR) (55, 56). Depending on local institutional policies and the specific clinical scenario, it may also be instituted in the field (mobile ECMO programs), at bedside in the ICU, or in the operating room (57). Full VA-ECMO support not only allows time to perform diagnostic and therapeutic interventions while maintaining appropriate hemodynamics and gas exchange, but also provides time for potential organ recovery. Multiple clinical trials are currently ongoing with the aim to address the potential clinical benefits of early VA-ECMO initiation in various patient populations (4, 25).

## Hemodynamic Aspects of VA-ECMO Support

VA-ECMO is used in the management of CS due to its capability to reduce myocardial work (pressure-volume area) while providing complete hemodynamic and respiratory support. Myocardial pressure-volume area can be thought of as the sum of myocardial potential energy and myocardial stroke work (58, 59). Both are thought to be increased profoundly in CS due to a vicious cycle of maladaptive neurohormonal and vascular mechanisms (8, 60).

In the typical VA-ECMO setup in CS, the venous inflow cannula drains blood directly from the vena cavae or the RA. This significantly decreases right ventricular (RV) preload, trans-pulmonary blood flow and, therefore, left ventricular end-diastolic volume (LVEDV) and pressure (LVEDP) (61–63). Thus, VA-ECMO likely promotes hemodynamic stabilization in the setting of CS and cardiac arrest via reduced LVEDV and LVEDP. It follows, then, that VA-ECMO has been shown to reduce stroke work in pre-clinical models of CS caused by acute myocardial infarction (64). The myocardial pressure-volume area and myocardial potential energy may be further reduced by the weaning of inotropic and vasopressor drugs once VA-ECMO support is instituted. These pharmacologic agents are known to increase myocardial oxygen consumption and left ventricular (LV) stroke work dramatically (59, 65).

The use of VA-ECMO also improves systemic perfusion. Typically, mean arterial blood pressure rises after VA-ECMO initiation while the high-volume venous displacement from the RA reduces central venous pressure. The systemic arterio-venous pressure gradient increases as a result, thereby enhancing systemic circulation. This may be particularly relevant to improving blood flow in organs with portal circulation, such as the liver and kidney (63). Fluid removal and relief of venous congestion can be further enhanced by splicing a continuous veno-venous hemodialysis machine (continuous renal replacement therapy; CVVHD) into the VA-ECMO circuit. By providing large volume oxygenated blood flow, organ perfusion can be supported irrespective of the intrinsic cardiac function. Importantly, the native right ventricular function is not as critical to the provision of systemic perfusion (as is the case with IABP and some pVADs) due to the lessened reliance on transpulmonary flow with VA-ECMO.

Despite the acknowledged benefits of VA-ECMO, there are still several critical gaps in the literature regarding the hemodynamic implications of prolonged VA-ECMO usage. Most notably, there is an absence of data using invasive ventricular catheterization to define how myocardial work and overall pressure-volume area is affected in the clinical (human) setting. Currently, most published pressure-volume loop data demonstrating the effects of varying levels of VA-ECMO support are based on computer simulations or animal experiments, rather than actual patient data (59, 66–68). Many of these studies used at least one fixed parameter (e.g., LV contractile strength) when performing their analysis. Yet, in real-life, these variables are interdependent and contractile strength will vary based on the Frank-Starling equation. Moreover, it is unclear how the hemodynamic responses on VA-ECMO support differ between patients with normal and depressed baseline LV ejection fraction, normal and dilated LV cavity and/or right ventricular dysfunction. Presumably, there is a diverse array of hemodynamic mechanisms in these HF sub-types, all of which remain largely uncharacterized *in vivo*.

The effect of retrograde arterial flow on LVEDV/LVEDP and LV unloading remains controversial and deserves special mention. Some commentators argue that the retrograde blood flow increases LV afterload by increasing mean arterial BP. This is thought to raise LVEDP, decrease stroke volume, reduce native cardiac output, and render a deleterious effect on LV performance (66, 68, 69). It is likely that this phenomenon more pertinent to patients with the complete lack of or minimal cardiac contractility, as opposed to patients that have preservation of LV function (63). Nevertheless, it is increasingly common to utilize one of the “LV venting” strategies, such as an IABP or Impella, despite unclear universal benefit (70). The device choice is often dependent of the center's experience and the benefit of upgrading from one strategy to another remains unexplored.

The populations in which venting devices offer a clear benefit remain largely uncharacterized. The hemodynamics of patients with different HF phenotypes are likely to respond differently to VA-ECMO support, thus creating a differential risk-benefit ratio for the addition of an unloading strategy. Patients with acute CS in the setting of severe, pre-existing HF and elevated left atrial pressure may be best suited for unloading. Moreover, patients with biventricular shock in whom the RV recovers before the LV, may also benefit from unloading. Under these circumstances, the RV may provide increased trans-pulmonary flow prompting a rise in LV preload, despite ongoing VA-ECMO support. The combination of increased preload and afterload may lead to an increase in the LV's myocardial oxygen consumption, thereby supporting the need for an unloading strategy.

## Common Indications for VA-ECMO Support

### Cardiogenic Shock Complicating Acute Myocardial Infarction

Despite the widespread use of early revascularization strategies, 6–10% of patients with acute coronary syndrome will progress to develop CS, representing 60–80% of all CS cases (12, 14, 15, 71). Myocardial ischemia and necrosis may continue following the index injury as the infarct extends circumferentially and toward the subepicardial regions. This prompts a further decline in cardiac function, increase in filling pressures, and excess oxygen consumption of the healthy residual myocardium. These, combined with reduced coronary perfusion pressure, initiate a vicious cycle until ~50% of the functional LV mass is lost and CS ensues. Initiating VA-ECMO early in this setting reduces cardiac work, myocardial oxygen consumption and improves coronary blood flow. Therefore, VA-ECMO may limit infarct extension and allow time for the hibernating myocardium to recover (72).

The in-hospital mortality of post-MI patients with CS approaches 70–80% with traditional management, including vasoactive agents and IABP (12, 16, 17). Several non-randomized trials have demonstrated a clear benefit of VA-ECMO support in this population. As a result, its use has increased over 5-fold between 2000 and 2010 in one report (73). In a single-center retrospective study of 98 patients with MI, early VA-ECMO cannulation was associated with an all-cause in-hospital mortality of 67.3%. Patients presenting with CS as well as cardiac arrest were included (74). In a single center, retrospective observational study, Pozzi et al. identified 56 post-MI patients who presented with evidence of CS and were supported with VA-ECMO for a mean of 8.7 days. Survival to hospital discharge reached 41.1 and 32.1% were alive after a mean follow-up of 38.0 ± 29.9 months (27). In another single center study from Korea, 20 patients with post-MI CS were initiated on VA-ECMO before proceeding with coronary revascularization. Although CPR was performed in 70% of the cohort before cannulation, the in-hospital survival rate reached 50% (75). Multiple other, relatively small studies from around the world have reported similar rates of successful VA-ECMO decannulation and hospital discharge in the setting of post-MI CS (10, 23, 24, 30, 33, 34, 76–78) (Table 2).

**Table 2:** Outcomes of VA-ECMO support stratified by the initial cause of cardiogenic shock.

**Indication for VA-ECMO support**	**Reported survival (%)**
Acute myocardial infarction	33.8–66.7
Cardiomyopathy	35.7–57.0
COVID-19 infection	0–36.6
eCPR	8.8–54.0
Fulminant myocarditis	60.0–74.0
Primary graft failure post heart transplantation	50.0–84.2
Massive pulmonary embolism	38.5–53.1
Cardiomyopathy in the setting of sepsis	59.8–75.0

*eCPR, extracorporeal cardiopulmonary resuscitation*.

Ventricular septal rupture (VSR) is a rare but dreaded complication of acute STEMI. It typically develops within 1–5 days after the STEMI and confers ~90% mortality (79) due to the rapid development CS. VA-ECMO may be an effective temporary hemodynamic support strategy to stabilize these patients. It can be instituted promptly and utilized as a bridge to definitive surgical management while allowing the friable myocardium surrounding the rupture site to mature (80, 81). A case series of three individuals with post myocardial infarction CS and VSR placed on VA-ECMO showed excellent results with decannulation achieved in all patients and 100% survival (82).

The timing of VA-ECMO cannulation is of paramount importance in this population. It should be initiated within 60 min of the recognition of refractory CS, especially if initial attempts at hemodynamic stabilization with fluid resuscitation and pharmacological agents fail (83). Preferably, MCS support should be established prior to proceeding with coronary interventions (28). The increased and early utilization of VA-ECMO in patients with post-MI CS is expected to translate into further improved clinical outcomes.

### Cardiogenic Shock Caused by Acute Fulminant Myocarditis

Acute fulminant myocarditis is a relatively uncommon, but severe condition characterized by the sudden and profound inflammation of the myocardium. Although the exact pathogenesis often remains obscure, myocyte edema and necrosis develop in response to various infectious and non-infectious triggers. The ensuing hypotension may progress to refractory cardiogenic shock within 2 days to 2 weeks of the initial insult. Owing to the profound hemodynamic instability and biventricular failure, escalating doses of vasoactive medications and IABP are often insufficient to maintain sufficient organ perfusion. VA-ECMO is an invaluable asset in the management of these patients. It may limit ongoing myocardial damage by providing prompt and effective circulatory support until the inflammatory storm subsides. Although VA-ECMO may serve as a bridge to durable left ventricular assist device (LVAD) or heart transplantation, full cardiac recovery is common within seven to 10 days in patients with fulminant myocarditis. With the exception of giant cell myocarditis, disease recurrence is uncommon and medical management is effective.

The available data also reflect a relatively positive prognosis in this population. In a multicenter, retrospective study of 57 patient with fulminant myocarditis, the mean duration of VA-ECMO support was 9.9 ± 19 days. 71.9% of patients were successfully discharged from the hospital and 5-years survival rate reached 65.2% (84). Another small, single-center study performed in Japan between 1991 and 2001 enrolled 14 patients with fulminant myocarditis requiring percutaneous VA-ECMO support for an average of 6.25 days. 71% of the cohort was weaned successfully and all of these had full cardiac recovery within 6–12 months (85). A study utilizing the ELSO database from 1995 through 2011 included 147 patients with a diagnosis of acute myocarditis who underwent ECMO support and showed a survival to hospital discharge rate of 61% (86). Many other groups have reported similarly high weaning and hospital discharge rates, establishing VA-ECMO as an extremely effective strategy for the management of patients with fulminant myocarditis associated with hemodynamic collapse (87–100) (Table 2).

### Acute Pulmonary Embolism/Right Ventricular Failure

The rate of hospital admissions for acute pulmonary embolism (PE) continues to rise and it remains one of the leading causes of cardiovascular death in the US (101, 102). Mortality reaches 80% in patients needing mechanical ventilation, 77% in those who require CPR within the first 24 h of admission and 37% in patients with syncope (103). Once the diagnosis is established, immediate risk stratification is critical. High-risk (massive) PE is characterized by: (I) Sustained systemic hypotension (systolic BP <90 mmHg for at least 15 min or requiring inotropic support with no other identifiable underlying causes, such as arrhythmia, sepsis or hypovolemia); (II) Clinical evidence of shock; III) Pulselessness or profound bradycardia (heart rate <40 BPM) (104, 105). Obstruction of 30–50% of the pulmonary vasculature in combination with vasoconstriction caused by thromboxane A_2_ and serotonin released from activated platelets lead to an acute increase in pulmonary vascular resistance (106, 107). As the unconditioned right ventricle (RV) is rarely able to generate a mean pulmonary artery (PA) pressure >40 mmHg in the acute setting, stroke volume decreases, the ventricle dilates and, ultimately, RV failure develops (108). The associated coronary hypoperfusion and myocardial ischemia lead to a further decline in RV function. These changes are critical as short-term mortality is driven primarily by the RV failure. In addition to the hemodynamic changes, respiratory failure is also common in patients with acute high-risk PE owing to the immediate development of ventilation-perfusion (V/Q) mismatch.

Most patients with massive PE and shock die within the first hour of presentation (109). Therefore, it is vital to initiate hemodynamic and respiratory support as early as possible after patient contact. Of the available MCS devices, peripheral VA-ECMO is the only system that can provide both and can be instituted within minutes in experienced centers. It allows rapid patient stabilization and therapeutic interventions to be performed, such as thrombolysis or thrombectomy. VA-ECMO removes blood from the RA in the veno-arterial configuration and, after oxygenation and CO_2_ elimination, returns it to the arterial system bypassing the pulmonary circulation. Therefore, it reduces RV strain, stabilizes the PA pressure, increases systemic perfusion and normalizes gas exchange.

To date, only a limited number of studies are available on the use of VA-ECMO in the setting of massive PE. These are mostly case reports and case series (110, 111) and no randomized clinical trials have evaluated the safety and efficacy of this approach. Overall survival rates are highly variable and depend on the definitive interventions used to manage PE, such as thrombolysis, surgical thrombectomy or heparin administration. In some reports, survival reaches 70% with good neurological function at discharge (Table 2). Cardiac arrest prior to VA-ECMO initiation and a lactic acid level exceeding 6 mmol/L was associated with worse outcomes (112–117). The recent European Society of Cardiology guidelines state that VA-ECMO may be considered, in combination with surgical embolectomy or catheter-directed treatment, in patients with PE and refractory circulatory collapse or cardiac arrest if appropriate expertise and resources are available (Class IIb, level of evidence: C) (118). Randomized controlled trials are needed to establish the clear benefit of VA-ECMO support in this population.

### VA-ECMO Use in the Setting of COVID-19-Associated Cardiogenic Shock

Coronavirus disease 2019 (COVID-19) was declared a pandemic by the World Health Organization on March 11, 2020. The causative virus, SARS-CoV-2 is highly infectious with a case fatality rate approaching 5.94% in the United States (119, 120). Although relatively rare, the most severe complications include acute respiratory distress syndrome, acute coronary syndrome secondary to coronary thrombosis or microembolism and stress-induced cardiomyopathy (121–127). SARS-CoV-2 affects most, if not all organs in the human body and the heart is no exception. In a series of 138 patients admitted with COVID-19 infection, the rate of acute cardiac injury was 7.2% (128). Another, smaller study documented an even higher rate of 17% (129). In both series, cardiac injury was defined by elevation of cardiac biomarker levels >99th percentile or the presence of new abnormalities on electrocardiography or echocardiography.

Given the prior use of VA-ECMO in patients with H1N1-associated myocarditis, several centers implemented VA-ECMO support for COVID-19-related CS. Given the extreme number of infections and limited resources, the Extracorporeal Life Support Organization (ELSO) has released guidelines on the contraindications for VA-ECMO use in this population (126, 130). These include, but are not limited to: advanced age, presence of any terminal disease, severe central nervous system injury, significant underlying comorbidities (such a dementia, liver failure, metastatic malignancy), severe multiorgan failure, severe peripheral vascular disease, “do not resuscitate” status, clinical frailty scale category ≥3, contraindications to anticoagulation, inability to accept blood products and ongoing CPR. The decision to proceed with VA-ECMO initiation should be made on a case by case basis after discussion with family and using a multidisciplinary team approach (131).

Recent reports suggest that only 5% of ECMO-supported patients for COVID-19 infection required VA configuration, while the need for VAV cannulation was reported in 6% (132, 133). As the severity of CS improves more rapidly than the respiratory failure, most patients on VAV-ECMO were ultimately converted to VV support for ongoing ARDS. Literature on patient survival requiring VA-ECMO cannulation for COVID-19-associated hemodynamic collapse remains scarce (Table 2). Further studies, such as the ExtraCorporeal Membrane Oxygenation for 2019 novel Coronavirus Acute Respiratory Disease (ECMOCARD) are warranted in this population.

In the case of respiratory failure and severe right ventricular dysfunction with preserved LV function, a veno-venous cannulation strategy with an oxygenator spliced into the circuit may be considered (Protek Duo oxyRVAD; Tandemlife, Pittsburgh, PA). A retrospective study by Mustafa and colleagues showed a mortality rate of 15% in 40 patients with most achieving freedom from ventilator care and ECMO support (134). Further studies are needed using this system in patients with severe COVID-19 infection.

### Extracorporeal Cardiopulmonary Resuscitation

VA-ECMO is increasingly utilized as a support strategy in the setting of out-of-hospital and in-hospital cardiac arrest. The provision of early extracorporeal cardiopulmonary resuscitation (ECPR) can maintain vital organ perfusion during and immediately after the arrest. In addition, ECPR provides full hemodynamic and respiratory support while reversible causes of the cardiac arrest are addressed and allows time for patients to recover from multi-organ failure (61).

Data regarding this approach has been available in the literature for over a decade. Survival rates for out-of-hospital cardiac arrest with ECPR use have varied widely from 7 to 45% (135–149). The disparity in outcomes seen in observational data is likely attributable to the broad heterogeneity of the study protocols. Some of these sources of heterogeneity include (I) the type of rhythm (shockable vs. non-shockable), (II) cannulation site (field, emergency room, or cardiac catheterization laboratory), and (III) intensive care unit strategies used in the post-arrest period. Moreover, there is a steep learning curve for the rapid, efficient, and safe initiation of peripheral VA-ECMO in the setting of cardiac arrest, particularly when CPR is ongoing.

Several observational studies from the Minnesota Resuscitation Consortium (MRC) support the use of ECPR strategy for select patients. Early data from the group described the feasibility of community-wide implementation of an ECPR approach (55). It was demonstrated that, through close collaboration with community emergency medical services, it is possible to facilitate rapid patient transfer to an ECPR hub where immediate VA-ECMO initiation and coronary revascularization is feasible. Accordingly, 50% of the patients enrolled in this protocol demonstrated survival to discharge with good neurologic function despite presenting with refractory ventricular tachycardia/ventricular fibrillation (VT/VF) arrest and ongoing CPR. Subsequent data from the group validated these survival results and suggested that rapid coronary revascularization is fundamental to improving outcomes and achieving high survival rates to discharge, owing to the incidence of underlying severe coronary artery disease in this population (150). This was further corroborated by a retrospective cohort study from the MRC where the ECPR approach was associated with improved rates of neurologically favorable survival to discharge compared to a matched cohort from the ALPS trial receiving standard advanced cardiac life support (ACLS) (151). Again, this is likely due to the ability of VA-ECMO to mitigate the severe and progressive metabolic derangements that occur with prolonged CPR. Collectively, these data from the MRC suggest that early VA-ECMO initiation combined with rapid coronary revascularization and an intensive care bundle promotes organ recovery, including cardiac function, following out-of-hospital cardiac arrest (152) (Table 2).

More recently, the MRC has published a single center randomized trial (Advanced reperfusion strategies for patients with out-of-hospital cardiac arrest and refractory ventricular fibrillation; ARREST) of 36 patients with out-of-hospital cardiac arrest due to refractory VT/VF. Patients were randomized to receive ECPR or standard ACLS on presentation. Patients in the VA-ECMO-facilitated resuscitation cohort had significantly higher in-hospital (43 vs. 7%) and post-discharge survival at 6-months (43 vs. 0%, *p* = 0.0063) (55). This was the first randomized clinical trial clearly demonstrating the benefits of a mature ECPR program. Several studies are currently planned or underway to invasively study the hemodynamic changes associated with VA-ECMO support (153–155).

### Other, Rare Indications for VA-ECMO Use in the Setting of Cardiogenic Shock

Other indications for VA-ECMO use include (I) *Sepsis in the setting of underlying cardiomyopathy*. Hemodynamic collapse may develop as the left ventricle is unable to augment cardiac output to counteract the severe vasodilation. Limited data has shown a benefit for VA-ECMO use in selected patients (6, 7, 156) (Table 2); (II) *Primary graft dysfunction following orthotopic heart transplantation*. Several studies have shown significantly improved outcomes when VA-ECMO is initiated early in this setting (11, 20, 157, 158) (Table 2); (III) *Obstructive shock*. Large intracardiac mass lesions, most commonly metastases, may limit blood flow across the cardiac valves. This may lead to severe hypotension and, ultimately, obstructive shock. Of the available MCS devices, VA-ECMO is the only option to support hemodynamics in this setting.

## Complications of VA-ECMO Support

Although peripheral VA-ECMO is a promising strategy that provides life support to patients with refractory CS, its use may be associated with potentially devastating complications. Some of these are preventable. Here, we review some of the common complications encountered while initiating or managing patients on VA-ECMO.

### Hemocompatibility-Associated Complications: Bleeding and Thrombosis

Bleeding is the most common complication reported in patients supported with VA-ECMO. In addition to access site bleeding, the risk of systemic hemorrhage is inherently increased in this population. Upper and lower gastrointestinal bleeding, hemopericardium, hemothorax, intra- and retroperitoneal hemorrhage and intracranial bleeding are the most frequent. It may be attributed to a combination of factors: (I) Acquired coagulopathy owing to blood exposure to artificial MCS surfaces, (II) Anticoagulation strategies used to reduce the risk of *ex vivo* thrombus formation, (III) Shear stress-associated platelet activation, (IV) Consumptive coagulopathy, (V) Constant activation of the fibrinolytic system, (VI) Systemic inflammatory response in the setting of CS and cardiac arrest, (VII) Infections and sepsis especially in the setting of prolonged support, and (VIII) Trauma associated with CPR and invasive procedures.

There is no clear consensus on anticoagulation strategy with VA-ECMO use and practice differs significantly between centers and individual patients. The risk of thrombosis and hemorrhagic complications must be balanced in the clinical context. Similar to our center, the most commonly reported strategy is the use of intravenous heparin for the duration of VA-ECMO support. However, the use of bivalirudin and novel anticoagulants have also been described (159). Adding to the controversy, optimal anticoagulant dosing remains unclear and needs to be individualized [prophylactic vs. therapeutic level; (159, 160)]. There is also emerging evidence that holding anticoagulation while on VA-ECMO may be safe in select patients and may decrease hemorrhagic complications and the requirement for blood transfusions without increasing mortality (161, 162). Regardless of the strategy and dosing selected, coagulation status must be monitored meticulously during VA-ECMO support. Various laboratory tests can be used depending on institutional protocols and the anticoagulant selected, such as activated clotting time (ACT), heparin anti-Xa level, activated partial thromboplastin time (aPTT), global thromboelastography (TEG), and prothrombin time (PT). Maintaining the platelet count above 50,000/mm^3^ and replacing coagulation factors as needed also reduces bleeding risk significantly.

Although thromboembolic complications have decreased in recent years with the introduction of biocompatible materials, they are still common and may have devastating clinical consequences, such as stroke (163, 164). In fact, embolic brain infarction has a reported prevalence of 1.7–15% with significant associated morbidity and mortality (165–168). Therefore, regular inspection of the circuit, including all connectors, is of critical importance. It is mandatory to continually monitor the pressure gradient across the oxygenator, the most common site for thrombus formation (169). Thrombosis at the pump head is rare but may lead to significant hemolysis and ultimately pump failure. Any thrombus beyond the oxygenator can cause systemic embolization as the blood is returned directly into the arterial circulation. Therefore, discovering a clot may necessitate the immediate replacement of the affected components. The most common etiology for thrombus development is blood-non-endothelialized extracorporeal circuit interactions that not only activates the coagulation pathway but also initiates a complement-mediated inflammatory response (170). Therefore, all patients are carefully anticoagulated using heparin or, less frequently, bivalirudin balancing the risk of bleeding and clotting. Heparin induced thrombocytopenia (HIT) is a relatively rare but highly prothrombotic condition. Monitoring platelet count on a regular basis is essential and further laboratory testing should be performed if any suspicion for HIT.

### Vascular Complications

The rate of access site complications is reported at around 20% and are mostly related to the urgent need to establish large-bore peripheral vascular accesses (5, 171). The spectrum of complications includes posterior vascular wall perforation, vessel dissection, pseudoaneurysm development, and thrombosis/embolic events. Patients are prone to large hematoma formation (intramuscular, retroperitoneal) even in the setting of minor vascular injury owing to the systemic anticoagulation employed for the VA-ECMO circuit. Most of these complications may be managed conservatively, while others warrant urgent endovascular or open surgical repair. The presence of peripheral artery disease poses an increased risk. The routine use of ultrasound and/or fluoroscopic x-ray guidance is recommended while obtaining vascular access as it allows precise target vessel visualization reducing the risk of injury (56).

Another serious vascular complication associated with peripheral VA-ECMO use is ipsilateral lower extremity ischemia. The clinical presentation often includes pallor, cool extremity, and gangrene development. Pain and neurological deficits may be difficult to assess owing to the sedation while patient is on VA-ECMO. A pooled analysis of 20 studies including 1,866 patients supported with VA-ECMO for CS or cardiac arrest reported a 16.9% (12.5–22.6%) incidence of lower limb ischemia; the risk of compartment syndrome or need for fasciotomy was 10.3% (7.3–14.5%). Lower extremity amputation was necessary in 4.7% (2.3–9.3%) of patients (172). Several risk factors have been identified to increase the risk of limb ischemia. These include younger age owing to the smaller femoral vessel size, female gender, the presence of peripheral arterial disease, difficult vascular access, and the use of larger bore cannulas (173–175). The routine use of a small anterograde reperfusion catheter has been shown to further reduce the risk of limb ischemia (174, 176). Ideally, it should be placed at the time of VA-ECMO initiation (174). At our center, heparin is often infused into the catheter according to the low intensity protocol to prevent thrombus formation and distal embolization. In addition, routine monitoring using near-infrared spectroscopy (NIRS) and doppler ultrasound is recommended in the clinical practice (177).

Access site infections may occur in 7–20% of patients with femoral VA-ECMO support (5, 174). Meticulous attention should be given to aseptic technique at the time of cannulation but this may be challenging at times given the emergency nature of the procedure that is often performed while CPR is in progress. Infections may range from local cellulitis to systemic bacteremia and sepsis and require appropriate antibiotic management.

### North-South (Harlequin) Syndrome

**Figure 1:**
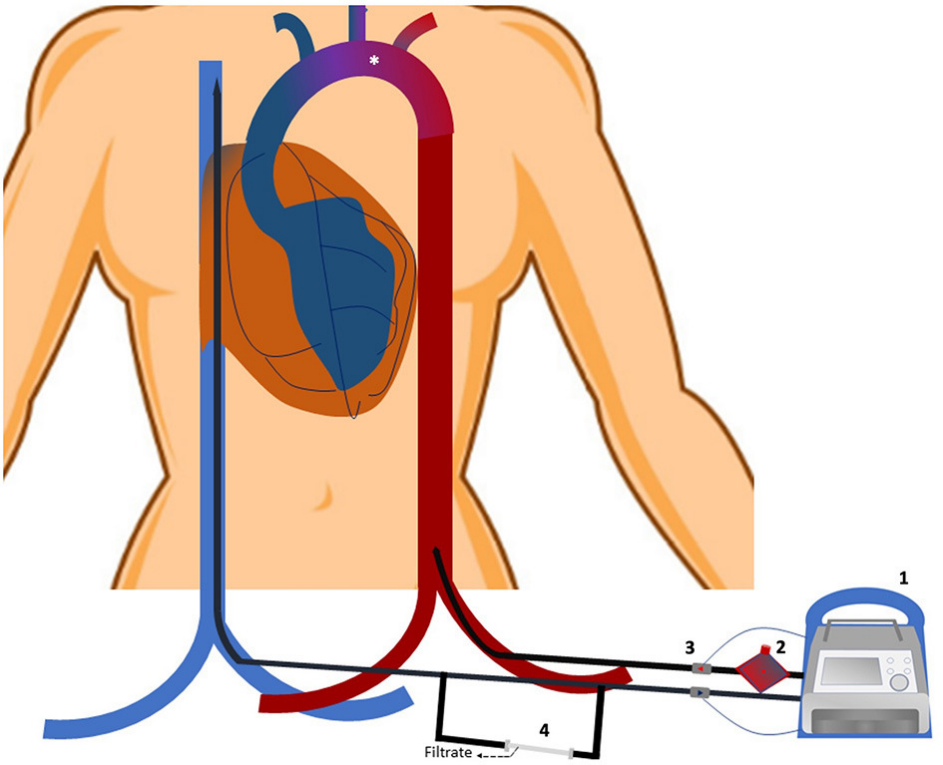
Veno-arterial extracorporeal cardio-membrane oxygenation (VA-ECMO) circuit and North South syndrome. A venous cannula is inserted into the superior vena cava/right atrium to drain deoxygenated blood by the extracorporeal pump (1). After passing through the “membrane lung (2),” oxygenated blood is returned into the iliac artery through the arterial cannula. Proximal (venous) and distal (arterial) sensors monitor circuit flow (3). A continuous hemodialysis machine may be spliced into the venous limb of the circuit if needed to provide renal replacement therapy (4). In situations when the left ventricle recovers pulsatility yet the pulmonary gas exchange remains inadequate, deoxygenated blood may be ejected into the ascending aorta. As the fully oxygenated retrograde flow provided by the ECMO circuit collides with the deoxygenated blood in the aorta, a mixing cloud forms (*). Its location is determined by the native cardiac function and the level of competing ECMO support. If undetected, ischemia of the organs perfused by the anterograde flow may develop.

North-South Syndrome is a complication unique to peripheral VA-ECMO (178). It may develop under circumstances when native cardiac function recovers pulsatility, yet pulmonary function remains inadequate. Unless the lungs are able to perform appropriate gas exchange, deoxygenated blood travels through the pulmonary circulation and into the LV. Given the native LV contractility, the deoxygenated blood is then ejected into the ascending aorta. As a result, a mixing cloud forms between the anterograde flowing deoxygenated blood and the fully oxygenated retrograde flow provided by the circuit (179) (Figure 1). The location of the mixing cloud depends on the native cardiac function and the level of competing ECMO flow. All organs perfused by the anterograde flow are at risk for ischemia, including the myocardium and the brain. Therefore, arterial oxygen saturation and blood gases should always be monitored using samples obtained from the right radial artery as the innominate artery is the first branch to receive deoxygenated blood from the proximal aortic arch. Further, near-infrared spectroscopy is a non-invasive tool developed recently to detect changes in regional tissue oxygenation and perfusion. Its routine use in patients supported with VA-ECMO may reduce the risk of hypoxic brain injury. If the differential cyanosis cannot be resolved by increasing the circuit flow, an additional cannula may be placed into the right internal jugular vein to achieve a hybrid configuration [veno-arterial-venous ECMO (VAV-ECMO)]. In this case oxygenated blood will be directed toward the right atrium by incorporating a “Y” connector into the arterial limb of the ECMO circuit. The oxygen rich blood will cross the pulmonary circulation thereby improving saturation in the proximal branches of the aorta (180).

### Acute Renal Failure

Acute renal failure is frequent in patients supported with VA-ECMO (55.6%) and is associated with increased mortality (181). Several factors may contribute to the renal injury including systemic hypoperfusion and hypotension prior to cannulation, systemic inflammatory response, hemoglobinuria in the setting of hemolysis, microemboli of the renal vasculature and kidney hypoperfusion due to dysregulation of the renin angiotensin aldosterone system (169, 181–184). Renal replacement therapy is required in 46.0% of VA-ECMO supported patients and can be initiated by splicing a CVVH machine into the circuit (172).

### Infections

Infections are one of the most common complications in patients supported with VA-ECMO with a reported prevalence between 9 and 65% (185–188). Access site infections are common and might be related, at least in part, to the challenges maintaining a sterile field during emergency cannulation while patient is critically ill, and possibly, receiving cardiopulmonary resuscitation. Other common infectious sources include the urinary tract in the setting of prolonged indwelling catheter use, the respiratory system and surgical wounds. Several investigators described a strong correlation between the duration of VA-ECMO support and the development of infections (188–190). In addition, recent evidence suggests that VA-ECMO use is associated with alterations in the innate and adaptive immune systems, further increasing the risk (191). Common pathogens include Staphylococcus Aureus (often methicillin resistant), non-lactose fermenting gram-negative bacilli and Candida (187, 189). Infections, especially when severe, are associated with a significantly increased mortality, morbidity, delay in weaning and circuit failure (187, 189, 192, 193). In addition to prevention, close monitoring for signs of infection is critical in all patients, as these may be subtle or masked by the effects of the ECMO circuit, hematologic, or metabolic changes.

### Patient Immobility and Alternative Cannulation Configuration

One disadvantage of prolonged hemodynamic support via the femoral approach is the need for patient immobility to reduce the risk of cannula kinking and dislodgement. In an alternative VA-ECMO configuration, the venous drainage cannula is inserted through the right internal jugular vein and oxygenated blood is returned into the subclavian or axillary artery using an end-to-side vascular graft. While this strategy allows for extended support while ensuring appropriate cerebral perfusion and, potentially, patient ambulation, ipsilateral arm hypoperfusion is reported in 20% of patients. While early detection of arm hypoperfusion and compartment syndrome may prove challenging in the setting of continuous blood flow and vasoactive medication use, it is essential to avoid limb ischemia.

### Other Complications

Another complications that is not necessarily related to VA-ECMO include hyperbilirubinemia (12.2%) (194). Monitoring for this and management according to standard ICU cares is critical.

## Weaning From VA-ECMO Support

Following a few days of full cardiorespiratory support, decannulation may be considered once the initial condition necessitating VA-ECMO improved or resolved and vasoactive medications are reduced to a minimum or off. Regular weaning trials are performed to assess the patient's hemodynamic response to incremental decrease in support. However, to date, the literature on VA-ECMO weaning strategies and timing is limited and is often driven by institutional experience (195–197). In addition, the reported definition of successful weaning varies broadly (195, 198–200). These factors, in addition to the differences in CS etiology, lead to reported weaning rates of 31–76% (201). Further studies are needed to identify the most successful VA-ECMO weaning strategies, stratified based on the etiology of the CS.

## Contraindications to VA-ECMO Use

While VA-ECMO represents a potentially lifesaving intervention for acutely unstable patients, absolute and relative contraindications should be considered. Absolute contraindications are few and, in general, include life expectancy <1-year, acute or preexisting conditions that are incompatible with recovery and VA-ECMO weaning (neurological injury, disseminated malignancy) or if individual patient goals-of-care are not compatible with such level of cardiorespiratory support. Relative contraindications include advanced age (>75 years), unrepaired aortic dissection as the retrograde high velocity flow may further propagate the dissection flap, severe aortic regurgitation as this may lead to progressive left ventricular distension, advanced peripheral vascular disease when peripheral cannulation is considered, and contraindications to systemic anticoagulation (2). Caution should be exercised in patients with prior mitral valve replacement as VA-ECMO can dramatically decrease trans-mitral flow thereby increasing the risk of thrombus formation.

Having an exit strategy from VA-ECMO support is critically important and should always be considered before cannulation. Lack of such strategy may be considered a contraindication for cannulation. Broadly, the goals of VA-ECMO may be divided into bridge to recovery or bridge to advanced heart failure therapies (such as LVAD placement or heart transplantation). Defining the goals is complex with a multitude of factors contributing, such as clinical reason for hemodynamic collapse, end organ function, age, patient wishes and values. In addition, the chance of meaningful recovery may be unclear at the time of cannulation. When possible, discussion should be held with patient, family and multidisciplinary team using best clinical judgement to define the exit strategy as early as possible.

## Discussion

The stagnant in-hospital mortality rates for CS over the past several decades has highlighted the need to develop increasingly granular risk stratification models and to introduce novel MCS strategies to improve outcomes for these patients.

In response to these critical needs, multiple steps have been taken. SCAI has published a novel classification schema for CS (Stage A-E) in 2019 (35). It was proved to be reproducible and to predict in-hospital mortality as well as 30-days patient survival with medical therapy alone and with a variety of MCS interventions (31, 202–205). Additionally, VA-ECMO has evolved to the point where it can be initiated within minutes by experienced clinicians and provides full cardiorespiratory support for several days. Therefore, this strategy enables the transfer of the sickest patients to experienced centers where additional diagnostic/therapeutic procedures may be performed while stable cardiorespiratory status is maintained by the VA-ECMO device. However, in times of global health crisis, such as during the COVID-19 pandemic, rationing the use of highly resource intensive therapies, like VA-ECMO, has to be considered. Complex clinical and ethical decisions must be made following the recommendation of multi-disciplinary triage committees that work alongside clinicians to facilitate effective and equitable allocation of scarce resources (206).

Ultimately, the combination of better risk stratification of CS and the emergence of novel MCS strategies may improve outcomes and survival in the most severe cases of CS (SCAI Stages C-E). Accordingly, European and US guidelines on the use of VA-ECMO in patients with CS are evolving and we anticipate updates in the near future as more data becomes available (2, 207–209). In the meantime, further prospective, randomized clinical trials are needed to expand the results of the ARREST trial and to evaluate the effects of VA-ECMO support on the survival of patients with CS of various etiologies.

## Author's Note

Cardiogenic shock leads to ~100,000 hospitalizations each year in the United States alone with a significant proportion of these patients dying during the index admission. While acute coronary syndrome remains the most common underlying cause, the incidence of cardiogenic shock due to other etiologies has been increasing in recent years. Veno-arterial extracorporeal membrane oxygenation (VA-ECMO) is the most advanced temporary life support system that can uniquely provide full hemodynamic as well as respiratory support. Our paper provides a comprehensive review on the epidemiology and evolving definition of cardiogenic shock, including the most recent classification system introduced by the Society for Cardiac Angiography and Intervention (SCAI). We discuss the system components, cannulation strategies and hemodynamic aspects of VA-ECMO support in the context of contemporary observational and randomized data. Subsequently, we summarize the most common indications, contraindications and complications related to VA-ECMO usage.

## Author Contributions

All authors listed have made a substantial, direct and intellectual contribution to the work, and approved it for publication.

## Conflict of Interest

The authors declare that the research was conducted in the absence of any commercial or financial relationships that could be construed as a potential conflict of interest.
